# Comparative study on the technique and efficacy of microscope-assisted MI-TLIF and naked-eye MI-TLIF in lumbar revision surgery

**DOI:** 10.1186/s13018-024-04591-5

**Published:** 2024-01-31

**Authors:** JiaHuan Zhang, YiFang Yuan, HaoRan Gao, Bo Liao, JiXian Qian, XiaoDong Yan

**Affiliations:** 1 Department of Orthopeadics, Tangdu Hospital, Fourth Military Medical University, No.569, Xinsi Road, Xi’an, 710038 Shaanxi China; 2 Xi’an Medical University, Xi’an, 710054 China

**Keywords:** Microscope, Minimally invasive transforaminal lumbar interbody fusion, Lumbar postoperative recurrence, Spinal minimally invasive, Lumbar revision

## Abstract

**Background:**

Lumbar revision surgery can be performed by simple lumbar nerve decompression or lumbar interbody fusion, including percutaneous endoscopic lumbar discectomy, transforaminal lumbar interbody fusion (TLIF), etc. However, lumbar revision surgery is very difficult in surgical operation. We sought to explore the technique safety and efficacy of microscope-assisted minimally invasive transforaminal lumbar interbody fusion (MI-TLIF) in lumbar revision surgery.

**Methods:**

Cases of postoperative recurrence following lumbar spine surgery (*n* = 63) treated from December 2016 to July 2021 were retrospectively analyzed, including 24 cases of microscope-assisted MI-TLIF (microscopic group) and 39 cases of naked-eye MI-TLIF (naked-eye group). The operation time, intraoperative blood loss, incision length, postoperative drainage, length of hospital stay, initial operation, and visual analog score (VAS) of low back and leg pain before and at 7 days and 3 months after the operation and the last follow-up were compared between the two groups. The Oswestry Dysfunction Index (ODI) and the Japanese Orthopaedic Association (JOA) scores before and after the operation and the Bridwell interbody fusion grades at 1 year were compared. The independent t tests, Mann–Whitney U tests, and Chi-square tests were used for analysis.

**Results:**

All 63 patients were successfully treated by operation and were followed up for an average of 31.5 ± 8.6 months (range 12–48 months). The two groups had no significant difference in sex, age, incision length, initial operation, or operative segment (*P* > 0.05). There was no significance in operation time, VAS score, ODI score, and JOA score of low back pain or Bridwell interbody fusion grade between the two groups (*P* > 0.05). Significant differences in intraoperative blood loss, postoperative drainage, and the lengths of hospital stay were observed between the two groups (*P* < 0.05). Cerebrospinal fluid leakage (*n* = 2), edema of nerve roots (*n* = 2), and incision infection (*n* = 1) were observed in the naked-eye group. There were no complications in the microscopic group, such as cerebrospinal fluid leakage, edema of nerve roots, and incision infection.

**Conclusion:**

Although microscope-assisted MI-TLIF and naked-eye MI-TLIF are both effective during lumbar revision surgery, microscope-assisted MI-TLIF brings less trauma, less bleeding, shorter postoperative hospital stay, and faster recovery. Unlike traditional surgery, microscope-assisted MI-TLIF provides a clear visual field, adequate hemostasis, and nerve decompression.

## Introduction

Lumbar degenerative disease refers to the physiological and pathological process of natural aging and degeneration of the lumbar spine, including lumbar disc herniation, spondylolisthesis, spinal stenosis, and so on. The therapeutic approach includes conservative and surgical treatment. At present, the main surgical methods are nerve decompression, fusion, and internal fixation [1]. However, a small number of patients experience recurrences after surgical treatment and need revision surgery [2, 3].

Postoperative recurrence of lumbar vertebrae results in clinical symptoms and imaging changes corresponding to the affected lumbar segments and lower extremities, which may be attributed to failed back surgery syndrome (FBSS) [4]. The etiology of FBSS has been extensively studied in the literature [5–7], including improper selection of patients, incorrect or incomplete diagnosis, wrong surgical procedures, failure to meet the surgical objectives (incomplete decompression, pseudoarthrosis, intraoperative injury, lumbar segment instability), progressive diseases. In relapse cases after lumbar spine surgery, revision surgery should be considered when the effect of conservative treatment is poor [8, 9]. Destruction of the normal anatomical structure during the initial operation increases the difficulty of the revision surgery.

Lumbar revision surgery involves decompression and fusion, while posterior lumbar fusion and fixation are more commonly used in conventional surgery. It is widely acknowledged that traditional open spine surgery may lead to muscle injury, laminectomy, ligament resection and nerve traction, and damage to the posterior structure of the lumbar spine and susceptibility to lumbar spine instability after surgery [10]. Due to extensive scarring and adhesions after the first operation, it is difficult to dissect with no clear anatomical marks, leading to significant tissue injury, and increased susceptibility of the nerve root and dura mater to injury.

Significant inroads of technology and equipment have been achieved in recent years with revolutionary achievements in the research and development of minimally invasive spinal surgery. It has been established that minimally invasive spinal surgery reduces intraoperative bleeding and wound infection and preserves paraspinal muscles and nerves to a large extent, greatly reducing surgical injuries and promoting the postoperative recovery of patients. At the beginning of the twenty-first century, Foley et al. [11] reported minimally invasive lumbar interbody fusion (MI-TLIF) via the intervertebral foramen approach. Compared with traditional surgery, this technique has the advantages of less trauma, less bleeding, shorter hospital stay, and early return to work, and it has been continuously applied and improved for clinical treatment [12]. However, in lumbar revision surgery, MI-TLIF has some shortcomings, such as unclear field of vision and poor lighting, which leads to difficulties in the treatment of scar adhesion and may lead to surgical complications. Microscopes have been widely used in lumbar degenerative diseases, lumbar tumors, and other surgeries. In this study, microscope-assisted MI-TLIF was used to reduce the difficulty of revision operation, ensure patient safety, and reduce the risk of nerve damage by using a more powerful light source and the microscope's magnified field of view [13, 14].

This study has the following purposes: (i) to analyze whether microscope-assisted MI-TLIF could compensate for the disadvantages of traditional surgery, such as unclear field of vision, poor lighting, significant tissue injury, and increased incidence of complications when applied in lumbar revision surgery; (ii) to explore the recovery of patients after operation, such as JOA score, VAS, and ODI; and (iii) to explore whether microscope-assisted MI-TLIF represents a better approach for lumbar revision surgery to provide a reference to assist surgeons during clinical practice.

## Materials and methods

### Study subjects

A retrospective analysis was performed on 63 cases of lumbar postoperative recurrence who were admitted to Tang du Hospital of Air Force Military Medical University from December 2016 to July 2021. The inclusion criteria were: (1) patients with established degenerative lumbar disease that underwent lumbar surgery; (2) patients with symptoms such as lumbar and leg pain having an impact on daily life and work, despite conservative treatment for 3–6 months; (3) imaging evidence of postoperative recurrence of lumbar vertebrae corresponding to patient symptoms and signs; (4) patients with surgical indications that provided consent for revision surgery; (5) the operative segment is a single segment; and (6) complete imaging and follow-up data available. The exclusion criteria were: (1) patients with an initial diagnosis of lumbar degenerative disease; (2) cases complicated with other types of spinal canal occupying lesions, lumbar infection, lumbar tumor, scoliosis, deformity, etc.; (3) patients with surgical contraindications or could not tolerate surgery. In the microscopic group, there were 9 males and 15 females, with a mean age of 52.9 ± 13.3 years (range 36–83 years). In the naked-eye group, there were 18 males and 21 females with an average age of 53.1 ± 13.3 years (range 26–76 years). This study was conducted in accordance with the principles of the Helsinki Declaration, and ethical approval was obtained from the IEC of the institution for National Drug Clinical Trials, Tangdu Hospital, Fourth Military Medical University (K202210-05).

### Operation method

#### Microscope-assisted MI-TLIF

**Fig. 1:**
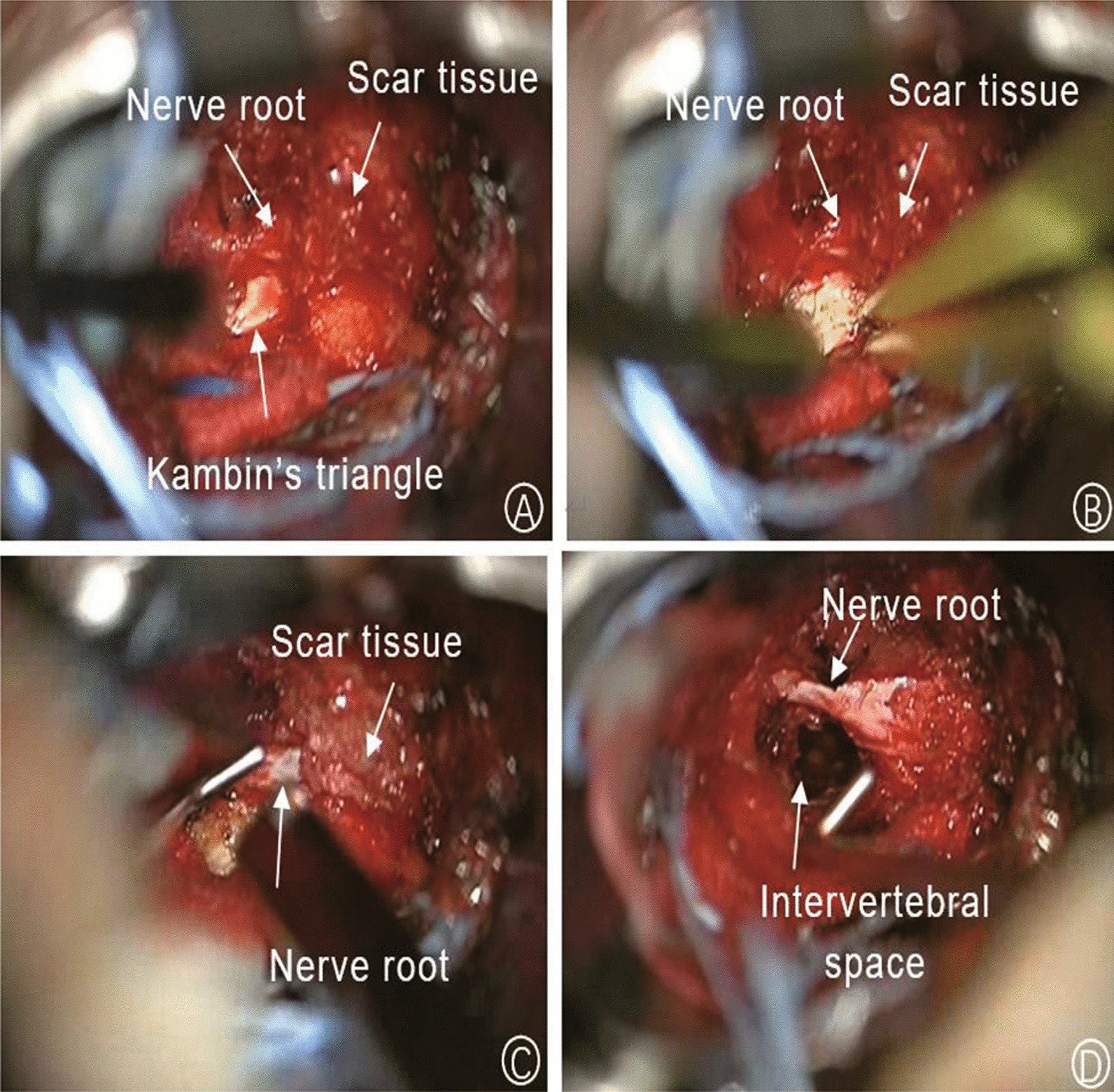
**A** Exposure of the Kambin's triangle to confirm the location of the nerve root; **B** proper hemostasis for a clear field of vision; **C** dissection of the scar and decompression of the nerve root; **D** exploration of nerve roots after decompression

After induction of general anesthesia, the patient was positioned prone. Body landmarks were identified using C-arm. The detailed procedure was follows: (i) The surgical area was routinely disinfected. A paramedian incision of 3–4 cm was made on either side, 2 cm lateral to the midline. After cutting the deep fascia, bluntly separating the paraspinal muscles and ensuring the facet joints can be touched, an expansion channel was installed with serpentine arm fixation, the light source of the channel is connected, and the upper and lower articular processes are partially excised. (ii) A microscope (OPMI Pentero) was used to magnify the surgical site for clear visualization of the intervertebral disc and surrounding nerves. Microscopically, a large number of scars adhered to dura mater and nerve roots, which were gently separated, excised, and fully hemostatic. The degenerated intervertebral disc was removed while ensuring satisfactory nerve root decompression (Fig. 1). (iii) The superior and inferior endplates were treated, and the disc space was flushed with saline. A bone graft obtained from the facets was packed in the disc space, followed by a cage filled with bone. (iv) After decompression and bone grafting, the retractor was removed from the working channel, and hollow pedicle screws were inserted into each pedicle along the guide wire. After the fluoroscopic position was satisfactorily confirmed, the pedicle screw rod system was installed. A negative-pressure suction device was inserted on one side of the incision, and the wound was closed layer by layer.

#### Postoperative treatment

Both groups were given postoperative analgesia, antibiotic prophylaxis, and nutritional support. Patients were advised to exercise both lower limbs to prevent venous thrombosis of the lower extremities. Twenty-four hours after the operation, the drainage device was removed when the drainage volume was less than 50 ml. The anterior and lateral lumbar vertebrae X-ray films were reexamined, and the patients were discharged after normal postoperative examination and good wound healing. Patients were advised to wear a back brace during exercise within 3 months after the operation and perform waist exercises.

#### Postoperative follow-up and evaluation

Outpatient follow-up was performed at 3 months, 6 months, 1 year and 2 years after the operation, and imaging data, including X-ray, CT and MRI of lumbar vertebrae in the anterior and lateral position, hyperflexion, and extension position, were reexamined.

The operation-related data were recorded, including operation time, length of incision, intraoperative blood loss, postoperative hospital stay, and complications.

Efficacy evaluation: Visual analog score (VAS) of low back pain was assessed before the operation, 7 days, 3 months after the operation, and at the last follow-up. Moreover, the Oswestry Dysfunction Index (ODI) was assessed before the operation, 3 months after the operation, and at the last follow-up. The Japanese Orthopaedic Association Score (JOA score) was evaluated before the operation, 1 year after the operation, and at the last follow-up. During postoperative imaging reexamination, the interbody fusion was evaluated according to Bridwell fusion grade.

#### Statistical method

Statistical analyses were performed with the statistical package SPSS (version 26.00). Continuous variables that followed an approximately normal distribution or normal distribution were expressed as mean ± standard deviation (SD); non-normal variables were reported as median (interquartile range). Then, independent t tests, Mann–Whitney U tests, Chi-square tests, and Fisher's exact tests were used to identify differences in clinical and radiological outcomes. A *P* value < 0.05 was statistically significant.

## Results

### General data of patients in both groups

There was no significant difference in sex, age, operation segment, or initial operation between the two groups (*P* > 0.05) (Table 1).

**Table 1:** Comparison of general data between the two groups of patients

Index	Microscopic group	Naked-eye group	Statistical value	*P*
Age(years)	52.9 ± 13.3	53.1 ± 13.3	*t* = − 0.066	0.948
Gender(M/F)	9/15	18/21	χ2 = 0.454	0.500
Operative segment			χ2 = 0.233	0.890
L3/4	2	4		
L4/5	15	22		
L5/S1	7	13		
Initial operation M/O	23/1	38/1	χ2 = 0.001	1.000

*M* Minimally invasive spinal surgery; *O* open spine surgery

### Comparison of treatment efficacy between two groups of patients

**Fig. 2:**
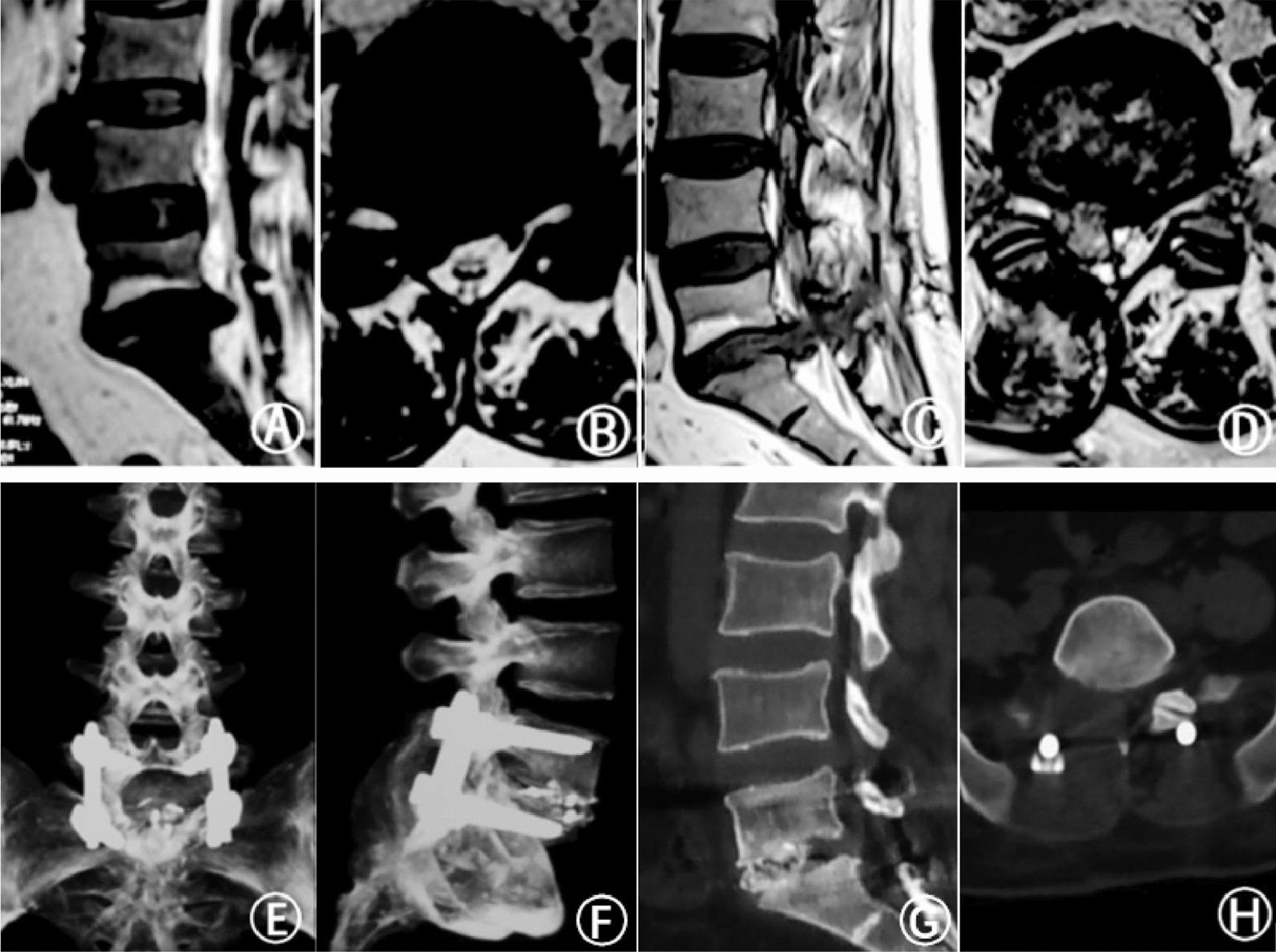
The patient was a 50-year-old female with low back pain and right lower limb pain and numbness for 2 years and was diagnosed with lumbar disc herniation. (AB)First preoperative MRI, patient underwent minimally invasive surgery. (CD)MRI before revision, the patient 's symptoms recurred, and the lumbar lesions and postoperative radiographic changes of lumbar spine can be seen in the figure. (EFGH) Lumbar spine imaging after revision surgery, bone graft fusion was successful

Surgery was successfully conducted in both groups. There were no significant differences in operation time, incision length, low back pain VAS score, ODI score, JOA, and Bridwell fusion evaluation grade between the two groups. However, the intraoperative blood loss, postoperative drainage, and length of hospital stay in the microscopic group were significantly lower than in the naked-eye group (*P* < 0.05) (Table 2). The VAS score, ODI score, and JOA score of low back pain in both groups were significantly improved after the operation (*P* < 0.05) (Table 3). A typical case is depicted in Fig. 2.

**Table 2:** Clinical indices of the two groups of patients after the operation(‾x ± s)

Index	Microscopic group	Naked-eye group	Statistical value	*P*
Operation time (min)	208.0 ± 69.6	204.0 ± 53.9	0.257	0.798
Total blood loss (ml)	250.0 ± 159.5	375.6 ± 223.7	− 2.400	0.019
Length of incision (cm)	8.2 ± 1.2	8.3 ± 1.3	− 0.275	0.785
Post-OP drainage (ml)	64.4 ± 37.1	168.3 ± 137.2	− 4.475	0.001
Hospital stay (days)	4.9 ± 1.9	6.4 ± 1.6	− 2.443	0.020

*Post-OP* Postoperative

**Table 3:** Clinical efficacy indices of the two groups of patients (x ± s)

Index	Microscopic group	Naked-eye group	Statistical value	*P*
*VAS-Back*				
Preoperative	5.5 ± 0.8	5.7 ± 0.7	− 1.268	0.210
7 days postoperatively	2.0 ± 0.7*	2.1 ± 0.6*	− 0.725	0.471
3 months postoperatively	0.6 ± 0.5*	0.8 ± 0.6*	− 1.819	0.074
Last follow-up	0.3 ± 0.4*	0.4 ± 0.5*	− 0.914	0.365
*VAS-Leg*				
Preoperative	6.6 ± 0.9	6.9 ± 1.0	− 1.248	0.217
7 days postoperatively	1.7 ± 0.7*	1.6 ± 0.6*	0.947	0.347
3 months postoperatively	0.5 ± 0.6*	0.7 ± 0.6*	− 1.135	0.261
Last follow-up	0.3 ± 0.4*	0.3 ± 0.5*	− 0.485	0.629
*ODI score*				
Preoperative	37.6 ± 2.3	36.7 ± 2.4	1.353	0.181
3 months postoperatively	25.0 ± 2.3*	24.4 ± 1.9*	1.134	0.261
Last follow-up	8.9 ± 1.6*	8.8 ± 1.2*	0.337	0.737
*JOA score*				
Preoperative	11.5 ± 1.7*	11.8 ± 1.8*	− 0.585	0.561
1 year postoperatively	22.2 ± 1.4*	21.8 ± 1.3*	1.069	0.289
Last follow-up	24.7 ± 1.2*	24.4 ± 1.4*	1.003	0.320
Bridwell(I/II)	18/6	27/12	χ2 = 0.242	0.623

^*^The same group was compared before the operation, *P* < 0.05

## Complication

Unlike the microscopic group, cerebrospinal fluid leakage (*n* = 2), edema of nerve roots (*n* = 2), and incision infection (*n* = 1) were observed in the naked-eye group. Two patients with cerebrospinal fluid leakage were given intraoperative wound layer-by-layer pressure suture, and postoperative position of head down and feet high was adopted, and drainage volume was closely observed. On postoperative day 5, the drainage volume decreased, the drainage tube was removed, and the patient was discharged smoothly; two cases of nerve root edema were treated by dehydration, improvement of circulation and nutrition; one patient developed wound infection 10 days after surgery and was given debridement and suture, 7-day antibiotic treatment, and regular dressing change, after which the wound healed.

## Discussion

In this study, microscope-assisted MI-TLIF was conducted through the multifidus and longissimus intermuscular approach. After gaining access to the scar-free or scar-less "safe triangle" area, dissection was conducted toward the medial scar area to reach the treatment target and can reduce the injury of paravertebral muscles and the destruction of posterior spinal structures. A clear of field with sufficient illumination was obtained under the microscope. Accurate hemostasis could be achieved during the operation. It was relatively easy to dissect the scar tissue, with few complications, less intraoperative bleeding, and postoperative drainage [15]. Microscope-assisted MI-TLIF has advantages over conventional surgery and MI-TLIF in lumbar revision surgery, especially on safety.

During revision operation, there are a large amount of scar tissue, and unclear anatomy in surgical field makes it difficult to release nerve root or dura adhesion, which increase the risks of dural tear and nerve root injury when releasing the scar. Therefore, the boundary of scar and normal tissue should be clearly distinguished for effective lysis and adequate hemostasis during operation.

### Characteristics of naked-eye MI-TLIF in lumbar revision surgery

In this study, MI-TLIF was used in both groups, which can avoid the original surgical scar in revision surgery and is widely used in clinics [16], but the naked MI-TLIF still has limitations. During treatment of severe peridural scars and adhesions, damage such as dural tear and nerve traction may occur due to limited visual field, limited operation space between the surgeon and assistant, and unclear surrounding anatomical fine structure. Accordingly, the risk of revision surgery is high, which may lead to injury of the dura mater, cerebrospinal fluid leakage, poor placement of pedicle screws, a large amount of bleeding, and even life-threatening. In this study, all operations were successfully carried out, although there were cases of cerebrospinal fluid leakage (*n* = 2), edema of nerve roots (*n* = 2), and incision infection (*n* = 1) in the naked-eye group. Due to the use of the intraoperative microscope, the operation of the microscopy group was better than the naked-eye MI-TLIF group in terms of safety profile.

For cases of cerebrospinal fluid leakage and nerve root edema in the naked-eye group, posterior decompression with discectomy of small incision, micro-endoscopic discectomy, and percutaneous endoscopic lumber discectomy, in addition, lumbar radiofrequency ablation, collagenase nucleolysis, and ozone nucleolysis were performed prior to revision surgery, regardless of whether the nucleus pulposus was removed. Scar adhesions always formed after the initial surgery, mainly because of inadequate hemostasis in intraspinal canals, regardless of whether physical or chemical methods were used. Inflammatory reactions, gradual fibrosis of granulation tissue, and scar formation occur in the surgical area, leading to the formation of scar tissue adhesions around the dura mater, nerve roots, and other tissues, and anatomical disorder in spinal canal. Revision surgeries have revealed that patients who underwent lumbar radiofrequency ablation, collagenase nucleolysis, and ozone nucleolysis had a higher incidence of scar adhesions. Furthermore, the initial operation and bleeding disrupted the anatomical structure, making operative area unclear during the revision operation. This lack of clarity always makes it very difficult to accurately separate the dura mater and perineural adhesion scars, increasing the risk of dura and nerve root damage.

When performing naked-eye revision surgery, it is crucial to pay more attention on the following mainly aspects of the operation to reduce complications. First, try to do better pre-hemostasis and control bleeding during operation using bipolar coagulation to get clear vision and minimize postoperative scar proliferation, and make sure the drainage is smooth without leakage. Second, clearly distinguish the anatomical structure including bone landmark, scar tissue, proliferative blood vessel, nerve root, and dura. Always keep in mind that starting decompression from the normal anatomical area to the abnormal to reduce leakage of cerebrospinal fluid. Third, it is not necessary to separate all the nerve root and dura from scars in the operating region, only the most serious compression should be removed. The more the separation and neurolysis, the more likely the nerve root and dura would be injured. Finally, try to make a better intervertebral space preparation. After revision of the discectomy and removal of the scars and osteophytes, it is recommended that a lumbar structural autograft of bone should be placed into the prepared disc space.

### The value of microscope-assisted MI-TLIF in revision surgery

Microscope-assisted MI-TLIF is increasingly used in the clinic, and the effect of the operation is more obvious than that of other operations [17–19]. Chen et al. used microscope-assisted MIS-TLIF to treat lumbar degenerative diseases, showing the advantages of less blood loss, short hospital stay, high accuracy of pedicle screw placement, and rapid recovery [20]. In this study, the intraoperative blood loss in the microscopic group was less in the naked-eye group (250.0 ± 159.5 ml vs. 375.6 ± 223.7 ml), suggesting less trauma and hemorrhage associated with the microscopic group. Moreover, postoperative drainage and postoperative hospital stay in the microscopic group were lower than in the naked-eye group (64.4 ± 37.1 ml vs. 168.3 ± 137.2 ml and 4.9 ± 1.9 vs. 6.4 ± 1.6 days). These results substantiated the advantages of less trauma and quick recovery after the operation in the microscopic group.

A clear visual field was obtained during microscope-assisted scar treatment, which enabled direct vision of hemorrhagic foci, while naked-eye scar treatment was associated with limited visual field, significant tissue injury, insufficient intraoperative hemostasis, and large amount of bleeding, which accounted for the significantly larger intraoperative blood loss and postoperative drainage volume in naked-eye group. Although good postoperative fusion outcomes were observed in the two groups, more accurate intervertebral treatment can be performed with the assistance of the microscope, with better exposure of the bony endplate, ensuring the success of interbody fusion.

Other advantages include a clear field of vision provided by adjusting the magnification and the focus, ensuring a good surgical field for a more precise operation. Moreover, for patients with severe lesions, dura mater, nerve roots, blood vessels, and other structures can be accurately identified in the process of nerve decompression, which reduces the risk of injury to important tissues during the operation. In addition, the chief surgeon and his assistant have the same surgical field of vision during the operation, significantly contributing to the operation's success. Furthermore, the surgical field can be displayed on a screen for clinical teaching with a clear view of the real anatomical structure of three dimension, providing more effective guidance on dealing with important structures. Finally, using an intraoperative microscope can reduce the frequency of lowering the head to ease tension on the cervical vertebra, although it should be borne in mind that it would take a long time to master the microscopic technique.

## Strengths and limitations

In this study, the microscope was used in minimally invasive lumbar surgery. Compared with MI-TLIF under naked eye, it was found that the microscope had the advantages of clear field of view, good depth sense, and high resolution, etc., which could play a certain guiding role in clinical work.

Given that this study is retrospective and the number of included cases is relatively small, our findings should be verified in future studies with larger sample sizes, longer follow-up time, and more evaluation indicators.

## Conclusion

Microscope-assisted MI-TLIF is an effective approach for lumbar revision surgery, which can reduce intraoperative injury and is conducive to the rapid recovery of patients after surgery.

## Data Availability

The datasets used and/or analyzed during the current study are available from the corresponding author on reasonable request.
